# Chemical Synthesis, Characterisation, and Biocompatibility of Nanometre Scale Porous Anodic Aluminium Oxide Membranes for Use as a Cell Culture Substrate for the Vero Cell Line: A Preliminary Study

**DOI:** 10.1155/2014/238762

**Published:** 2014-01-21

**Authors:** Gérrard Eddy Jai Poinern, Xuan Thi Le, Mark O'Dea, Thomas Becker, Derek Fawcett

**Affiliations:** ^1^Murdoch Applied Nanotechnology Research Group, Department of Physics, Energy Studies and Nanotechnology, School of Engineering and Energy, Murdoch University, Murdoch, WA 6150, Australia; ^2^Animal Health Laboratories, Animal Virology, Department of Agriculture and Food, 3 Baron Hay Court, Kensington, WA 6150, Australia; ^3^Department of Chemistry, Curtin University of Technology, Bentley, WA 6102, Australia

## Abstract

In this preliminary study we investigate for the first time the biomedical potential of using porous anodic aluminium oxide (AAO) membranes as a cell substrate for culturing the *Cercopithecus aethiops* (African green monkey) Kidney (Vero) epithelial cell line. One advantage of using the inorganic AAO membrane is the presence of nanometre scale pore channels that allow the exchange of molecules and nutrients across the membrane. The size of the pore channels can be preselected by adjusting the controlling parameters of a temperature controlled two-step anodization process. The cellular interaction and response of the Vero cell line with an in-house synthesised AAO membrane, a commercially available membrane, and a glass control were assessed by investigating cell adhesion, morphology, and proliferation over a 72 h period. The number of viable cells proliferating over the respective membrane surfaces revealed that the locally produced in-house AAO membrane had cells numbers similar to the glass control. The study revealed evidence of focal adhesion sites over the surface of the nanoporous membranes and the penetration of cellular extensions into the pore structure as well. The outcome of the study has revealed that nanometre scale porous AAO membranes have the potential to become practical cell culture scaffold substrates with the capability to enhance adhesion and proliferation of Vero cells.

## 1. Introduction

Anodization of aluminium (Al) is an electrochemical process that changes the surface chemistry of the metal. During this oxidation process, which is carried out in a polyprotic acid (e.g., oxalic, phosphoric, or sulphuric acid), the resultant anodic oxide layer formed contains a disordered array of pores. However, investigations by Masuda and Fukuda in 1998 revealed that a highly ordered hexagonal structure was only possible under specific anodizing parameters and the resulting oxide layer followed a self-organized growth mechanism [1]. It was during the long anodization periods (up to a maximum of 160 hours) used throughout their studies that the pores were able to self-adjust from their random initiation sites. The ordered pore positions were only seen at the metal/oxide interface after the barrier layer was removed. The initial pore sites seen on the surface of the oxide/electrolyte interface were the result of the random nucleation sites produced during the early stages of oxide formation [2]. Further refinement to improve the pore ordering during the two-step anodization process was carried out by Masuda and Satoh which resulted in the process producing straight, parallel, and densely packed hexagonally arrayed pore channels from the metal/oxide interface to the oxide/electrolyte interface [3]. The first step in this optimised technique is a long anodization period, which is used to form a highly ordered pore array at the metal/oxide interface. Removing the oxide layer to reveal a highly periodic and indented landscape covering the surface of the Al substrate follows this. These indentations form the initiation sites for the pores formed subsequently in the second anodization step [4]. During the second step, a densely packed, highly ordered pore array is produced [5, 6]. It was this improved technique of fabricating nanometre scale structures in the oxide layer that rekindled interest in using anodic aluminium oxide (AAO) membranes as a potential template for the manufacture of nanometre scale materials [7, 8], biological/chemical sensors [9, 10], filter membranes, [11] and medical scaffolds for tissue engineering [12–14].

For cells anchoring onto a solid substrate, cellular response and function depend on the surface characteristics of the substrate. Therefore, cell-substrate interactions are of fundamental importance since the attachment of the cell to the substrate is necessary for cell viability and growth. Cell adhesion to a substrate surface is of critical importance since it is a precursor to cell spreading, growth, migration and proliferation. Thus, when culturing cells, the surface environment of the substrate can have a significant influence on cell activity, adhesion, morphology, and proliferation [15]. Most cells are in the micrometre range; however, their component structures and environment are in the submicrometre to nanometre range. The nanometre scale is a very important factor, since the molecular building blocks of life such as proteins, carbohydrates, nucleic acids, and lipids are all at this scale. This is especially important since the interaction between cells and proteins mediates a substrate surface. The proteins are either adsorbed from the culture medium or secreted by the cultured cells. The mechanisms behind the adhesive attachment of an anchorage-dependent cell, the influence of the physical structure and surface chemistry of the surface in this interaction, and the influence of protein mediation are yet to be fully explained. Furthermore, cell functions such as migration, proliferation, and the production of the extracellular matrix (ECM) are all surface chemistry, surface structure, and protein dependent [16]. When a substrate is immersed in a culture medium, protein adsorption is dependent on surface properties such as surface charge, surface chemistry [17], surface density of cell-binding ligands [18] wettability [19], and nanometre scale topography [20]. These properties all play an important role in promoting the cell-substrate interaction, which ultimately decides the effectiveness of the substrate as a compatible biomaterial. It is this complex relationship between cell, surface chemistry, and nanometre scale surface topography that has produced considerable interest in recent years. A key factor that has come out of this recent interest is the potential ability of nanometre scale topography to mimic components of the ECM and promote a favourable adhesive response from the cell [21]. The ability of cells to sense nanometre scale topographical features in their environment has been shown by a number of researchers. Dalby et al. was able to show that filopodial extensions from fibroblast cells were capable of sensing topographical features as small as 10 nm [22]. While in a similar study by Nguyen et al., the response of smooth muscle cells to various nanometre size topographical features was successfully demonstrated [23].

The unique properties of AAO membranes have also been demonstrated as a promising biomaterial for potential tissue engineering applications [24]. Numerous studies on cell types such as hepatic cells [25, 26], neuronal cells [27], fibroblasts and keratinocytes [28], osteoblasts [29], smooth muscle cells [23], and stem cells [30] have all shown that an AAO membrane can be effectively used as a culture substrate. The results of these studies suggest that both the porous structure and topographical features of the membrane are influencing cellular behaviour. Therefore, the aim of the present study was to investigate the viability of using an engineered AAO membrane as a cell substrate for culturing the *Cercopithecus aethiops* (African green monkey) Kidney (Vero) epithelial cell line for potential tissue engineering applications. The cell line was cultured on two different types of nanometre scale porous AAO membranes, each with a fixed pore sized topography, with laboratory grade glass slides being used as the control substrate. The first membrane was manufactured in-house, while the second was a commercially available membrane (Whatman Anodisc 25, 0.1 *μ*m). Both membranes had a mean pore diameter of 100 nm but had different interpore spacing and surface roughness. The cellular response of the cell line to both porous membranes types and the glass control surface was evaluated over a 72 h period using a cell proliferation assay. Cell adhesion and morphology on all three substrates was investigated using optical microscopy (OM), field emission scanning electron microscopy (FESEM), and atomic force microscopy (AFM).

## 2. Materials and Methods

### 2.1. Materials

All chemicals were purchased from Sigma-Aldrich (Castle Hill: NSW, Australia) and used without further purification. Milli-Q water (18.3 MΩ cm^−1^) was used in all aqueous solution preparations and was produced from a Barnstead Ultrapure Water System D11931 (Thermo Scientific, Dubuque, IA). The 99.99% pure aluminium foil (0.25 mm thick) was supplied by Alfa Aesar (USA) and the anodisc membrane (diameter 25 mm, pore size 0.1 *μ*m) used for comparative purposes was supplied by Whatman Anopore (UK).

### 2.2. Fabrication of In-House Nanoporous AAO Membranes

**Figure 1:**
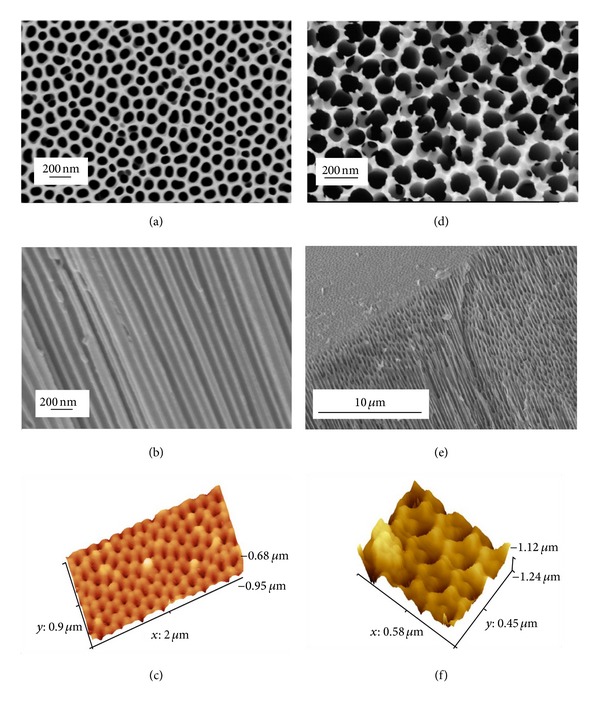
In-house AAO membrane: FESEM micrographs of (a) surface view, (b) cross-sectional view, and (c) AFM image of surface. Whatman anodisc membrane: FESEM micrographs of (d) surface view, (e) cross-sectional view, and (f) AFM image of surface.

Fabrication of the in-house membranes begins with a 100 mm square aluminium (Al) high purity (99.99%) sheet, 0.25 mm thick supplied by Alfa Aesar, USA, being cut into 50 mm × 20 mm strips. The strips are then placed into a tube furnace and annealed in a nitrogen atmosphere at 500°C for 5 hours to initiate recrystallisation and release any mechanical stresses in the strips. After annealing, the strips were degreased in acetone and then etched in a 3.0 M sodium hydroxide solution for 5 minutes before being thoroughly washed in Milli-Q water. The strips were then dried before a thin layer of polymer was applied to one side of the strip. Once the polymer had set, the strip was ready for the first step of the two-step anodization procedure. During the first step, each strip was anodized using a voltage of 60 V in an electrolyte solution consisting of 0.3 M oxalic acid for 5 hours. At the end of the first anodization step, the resulting thin oxide layer formed on the nonpolymer coated side of the strip was removed from the substrate by immersion in a stirred acidic solution composed of phosphoric and chromic acid (70 mL/L and 20 g/L, resp.) at 60°C for 1 hour. This is an important stage of the process, since it selectively removes the first oxide layer and exposes a highly periodic and indented landscape covering the surface of the Al substrate. These indentations form the initiation sites for pores formed in the second anodization step [4, 6]. The second anodization step is performed under the same conditions as the first step, except that the period of anodization is only for 3 hours. During the second step, a regular, honeycomb array of nanometer sized pores is formed across the surface of the oxide layer. After the second anodization, the pores were then widened by chemical etching in a 5% solution of phosphoric acid at 35°C for 15 minutes. Then a thin layer of Acrifix 192 was applied to the anodized side of the Al strip. This serves as a physical support for the membrane during the removal of the Al substrate using an acidic solution mixture composed of 0.1 M copper chloride and 7% hydrochloric acid. Following the removal of the Al substrate, the barrier layer was removed from the membrane by etching in a 0.3 M solution of phosphoric acid. The final etching stage results in the complete dissolution of the barrier layer and the acrylic support leaving an off-white coloured oxide membrane. The final stage in producing the scaffold membrane is the sterilization step, which involves immersing the membranes in a 30% solution of hydrogen peroxide at 60°C for 15 minutes. This was followed by quickly dipping the membrane into a solution of Milli-Q water for 10 seconds to remove any hydrogen peroxide from the membrane surface and then it was placed under ultraviolet light for 2 h. The membranes are then placed in airtight containers, wrapped in Al foil, and stored for future use.  Figure 1 presents a field emission scanning electron microscopy micrograph of a typical AAO membrane fabricated in-house using the two-step anodization procedure.

### 2.3. Characterization of Materials and Cells

The in-house fabricated nanometre scale porous AAO membranes and Whatmann Anodisc membranes were examined using field emission scanning electron microscopy (FESEM) and an atomic force microscopy (AFM). The FESEM micrographs were taken using the Zeiss Neon 40 EsB FIBSEM (Carl Zeiss, Oberkochen, Germany) located at the Centre for Materials Research (CMR) at Curtin University of Technology. The field emission electron gun provided both high brightness and high resolution (0.8 nm). Micrographs were taken at various magnifications ranging from 2 to 5 kV using the SE2 and InLens detectors. Samples were mounted on individual substrate holders using carbon adhesive tape before being sputter-coated with a 2 nm layer of platinum to prevent charge build-up using a Cressington 208HR High Resolution Sputter coater. The AFM images were taken using the Digital Instruments Dimension 3100 AFM set for Tapping Mode. The AFM tips used were supplied by Nanoworld innovative technologies and the silicon SPM sensor, (noncontact mode) was supplied by NCH-W PointProbe (thickness 4 *μ*m, width 30 *μ*m, and length 125 *μ*m). The AFM's force constant was 42 N/m and its resonance frequency was 320 kHz. Optical microscopy (OM) was used throughout the cell studies to examine cell-membrane interactions such as attachment, migration, and proliferation. An Olympus BX51 compound microscope (Olympus Optical Co. Ltd., Tokyo, Japan) was used for all optical studies and photographs were taken using the DP 70 camera attachment.

Before optical microscopy investigation, cells adhering to each respective membrane were fixed using a 1 : 1 solution of acetone and methanol. The cells were then stained using an aqueous solution containing 1% Fuchsin acid. After 1 h, the excess stain was rinsed off the membranes using Milli-Q water. After the membranes had dried, they were then mounted onto microscope slides ready for optical microscopy investigation at various magnifications (4x, 10x, 20x, and 40x). Samples for AFM analysis were mounted onto double-sided adhesive tape, which was then mounted onto the AFM stub ready for imaging. Before FESEM observation could be carried out, the cell/membrane samples needed to be washed in increasing concentrations of ethanol prior to being sputter-coated. Initially the cell/membranes were washed in a 30% solution of ethanol several times before being allowed to soak for 15 minutes in the ethanol solution. At the end of this period, sequential drying of the samples using progressively increasing concentrations of ethanol washes (2 washers of 50%, 70%, 80%, 90%, and 95%) was carried out until finally being washed in a solution of 100% ethanol for 30 minutes. Following the ethanol washing procedure, the samples were then treated with a 50 : 50 solution of ethanol : amylacetate for 30 minutes. This was then followed by 2 immersions in amyl acetate over a period of 1 h before being placed into a critical point dryer. Finally, the dried cell/membrane samples were mounted on FESEM stubs before being sputter-coated with a 2 nm layer of platinum metal for imaging purposes. The samples were then ready for FESEM investigations.

### 2.4. Cell Culturing and Growth on Membranes

#### 2.4.1. Cell Seeding and Culture

The cell line used in this *in vitro* study was the *Cercopithecus aethiops* (African green monkey) Kidney (Vero) epithelial and was supplied by the Animal Health Laboratories, Animal Virology, Department of Agriculture and Food, 3 Baron Hay Court, Kensington, Western Australia, Australia. The cell culturing protocol was carried out in accordance with the Animal Health Laboratories procedure VIW-17 using a Cell Growth Medium 199 (Sigma-Aldrich) and 10% fetal calf serum (FCS) (Virology Laboratory procedure VIW-17, Animal Health Laboratories, Animal Virology, Department of Agriculture and Food, 3 Baron Hay Court, Kensington, Western Australia, Australia). A standard cell culturing procedure used in previous AAO cell line studies by the authors is presented and discussed in [14].

### 2.5. Cell Adhesion Studies

Both types of nanoporous membranes (in-house and Whatmann Anodisc) were used without surface treatments, other than sterilization along with the glass controls prior to cell seeding. The cell adhesion studies started with making up a series of sample sets. Each sample set consisted of adding 2 samples of each nanomembrane type and 2 glass controls to a 6 well culture plate (Number  657-160) supplied by CellStar Greiner Bio-One, Germany. A sample run consisted of 4 samples sets, each set corresponding to time intervals of 4, 24, 48, and 72 h. Then a 3 mL solution of Vero cells (approximately 3 × 10^5^ cells/mL) suspended in culture medium and 10% FCS were transferred to each well of the culture plate using a pipette. The well plates were then gently oscillated to disperse the cells and then incubated at 37°C under a 5% CO_2_ atmosphere. At the end of the first hour, the first well plate set was removed from the incubator and the individual membranes and glass controls were fixed using a 1 : 1 solution of methanol : acetone. After 10 minutes, the membranes and glass controls were removed from the fixing solution and allowed to air dry at room temperature. Then the cells on both membranes and glass controls were stained using an aqueous solution containing 1% Fuchsin acid. After soaking the samples for 1 h, the excess stain was rinsed off Milli-Q water and allowed to air dry at room temperature. After the samples had dried, each sample was mounted onto a microscope slide before a cover slip was added. The samples were then ready for optical microscopic investigation. The procedure was then repeated for the 24 h, 48 h, and finally the 72 h time periods. Furthermore, the complete cell adhesion procedure for the 4, 24, 48, and 72 h periods was carried out in triplicate to ensure consistency in the study.

### 2.6. Cell Proliferation Studies

The number of viable Vero cells proliferating over the surface of each respective membrane and glass control was quantified over a 72 h period using a standard cell counting technique. The assay procedure consisted of overlaying a grid onto each photographic image of a random location on a substrate and then counting the number of cells present in that area. A minimum of 10 random locations per substrate was examined and then the mean ± standard deviation was calculated for the cell number on that particular substrate.

### 2.7. Statistical Analysis

The frequency and size of the particular surface features such as pore diameter, pore density, and interpore distance were determined by counting and physically measuring the size of the features found within 10 randomly selected 1 *μ*m square grids. From this analysis the mean ± standard deviation of each surface feature was calculated.

## 3. Results and Discussions

The structure and surface topography of both AAO membranes was examined using both (FESEM) and AFM. The in-house AAO membrane was synthesised using an optimised two-step anodization process. The process is straightforward and economical and produces highly reproducible membranes. It also permits the selection of material properties such as membrane thickness, pore size, interpore spacing, and pore density. The second membrane type examined was a commercially available alumina membrane supplied by Whatmann Anodisc. The present study found that the nanometre scale porous structure of both membranes had a surface topography that was beneficial for promoting both cell adhesion and proliferation. The study also investigated using the membranes as a viable of cell substrate via a cell proliferation assay, which was performed over a 72 h period.

The surface investigation of nanometre scale topographical features of both the in-house and commercial membranes was carried out using FESEM and AFM techniques. The in-house AAO membrane had a surface terrain composed of highly ordered, closely packed hexagonal arrays of uniformly sized pores. The surface landscape was smooth and undulating, with ordered pore domains tessellated across the entire surface as shown in Figure 1(a). Inspection of Figure 1(a) reveals the presence of occasional nonordered pores between the pore domains that are formed by point defects, dislocations, and grain boundaries in the original Al substrate [31, 32]. A survey of the membrane surface revealed a pore density of 53 ± 3 pores/*μ*m^2^ (mean ± std), a mean pore diameter of 104 ± 12 nm, and a mean interpore distance of 150 ± 14 nm. The consistency of the interpore distance can be seen in Figure 1(b), which presents the nanometre scale channels that traverse the membrane. The consistent geometry and narrow pore size distribution was further highlighted in the AFM images. A typical AFM image of the in-house membrane is presented in Figure 1(c) and confirms the presence of uniformly distributed pore arrays covering the landscape. The consistency of the nanometre scale pore structures present in the membrane clearly demonstrates the effectiveness of the two-step anodization process in producing a highly regular, closely packed, and uniform array of pores. The consistency and reproducibility of the two-step anodization process were extremely important since the subsequent *in vitro* cell studies needed to be carried out on membranes with a uniform surface topography.

The second type of AAO membrane used throughout the study was the commercially available Whatman Anopore (Anodisc) membrane [33]. The membrane is composed of a high purity alumina matrix containing a honeycomb based pore structure. The pores are circular in shape and have a nominal pore size of 0.1 *μ*m and a membrane thickness of 60 *μ*m.

The nanometre scale channels traverse the membrane (Figure 1(e)), which makes it ideal for a wide range of laboratory based filtration applications [33]. However, the manufacturer's specification contains no specific information regarding the type of synthesis process used to produce their membranes. Analysis of the Anodisc membrane revealed a consistent pore geometry and distribution, with no apparent pore domains present. The mean pore diameter was calculated to be 120 ± 45 nm, with the mean interpore distance of 0.32 *μ*m. The interpore distance was calculated from the nominal density of pores present in the membrane. X-Ray diffraction studies by Fisch et al. found a mean interpore distance of around 0.37 *μ*m for a typical Anodisc membrane [34]. The most interesting feature of the Anodisc membranes was the inconsistency in their pore wall thickness, which varied across the membrane. Another feature was the very rough edges of the pore walls, which protruded up from the membrane surface as shown in Figure 1(d). Overall, the surface landscape of the Anodisc membrane is very rough compared to the in-house fabricated AAO membranes, which tend to be smooth and undulating, with more refined nanometre scale topography. The greater surface roughness present in the Anodisc membrane was further enhanced by the AFM analysis which revealed the saw-like pore walls protruding from the membrane surface as shown in Figure 1(f).

**Figure 2:**
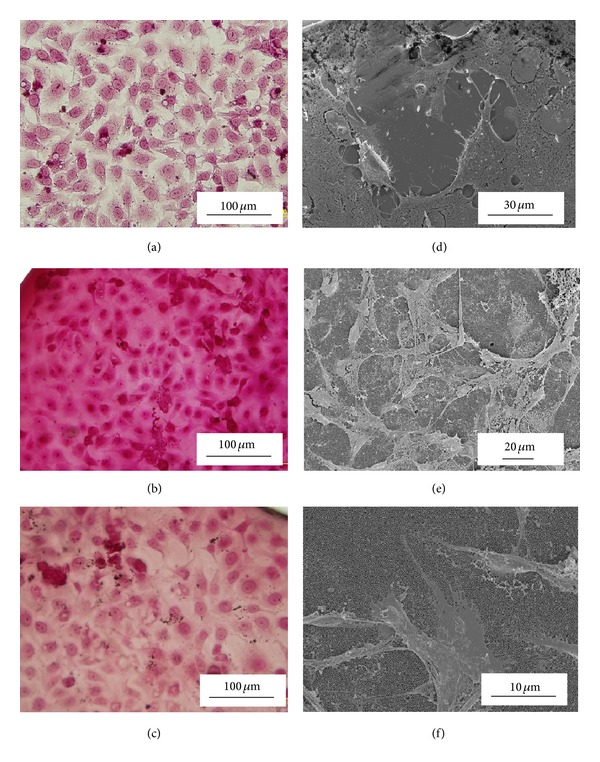
Optical Microscopy of Vero cells (a) glass, (b) Whatman anodisc membrane, and (c) in-house AAO membrane. FESEM Micrographs were taken at various magnifications ranging from 2 to 5 kV: (d) glass, (e) Whatman anodisc membrane, and (f) in-house AAO membrane.

**Figure 3:**
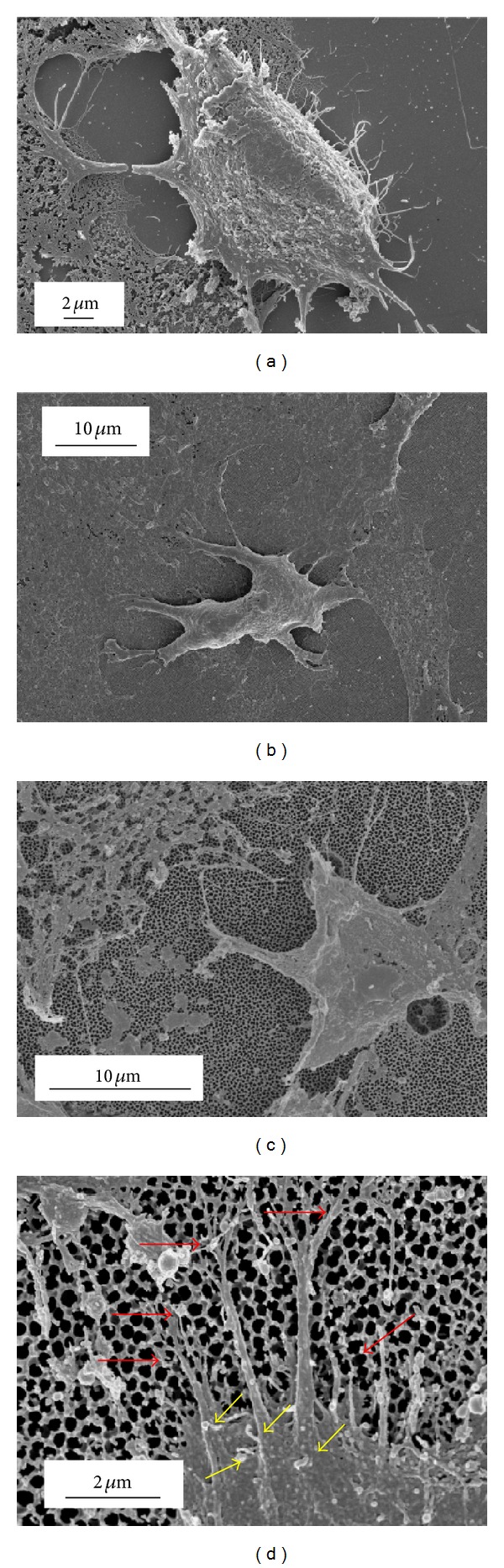
FESEM micrographs of cell attachment showing filopodia (a) glass substrate, (b) Whatman anodisc membrane, (c) in-house AAO membrane, and (d) enlargement of cell showing microvilli and filopodia on Whatman Anodisc membrane.

Optical microscopy observations carried out after 24 h of cell cultivation on both membrane types and the glass control revealed good cell adhesion and wide spread coverage over the entire substrate surfaces; see Figure 2. Closer examination of Figures 2(a), 2(b), and 2(c) reveals that the cells cultured on the nanoporous membranes are comparable to those cultured on the glass control substrate. The FESEM micrographs taken of the three substrates (Figures 2(d), 2(e), and 2(f)) also reveal that the Vero cells are distributed over the entire surface of each substrate and that the cells are actively generating ECM. The presence of the ECM confirms that the cells are actively interacting with the ECM and the substrate surfaces. FESEM micrographs taken of specific cells on each substrate reveal the presence of filopodia at the cell boundary; see Figure 3. The filopodia spread out over the surface of all three substrates. In the case of the AAO membranes there is evidence that the cells are using the pores as anchorage points. Furthermore, the FESEM micrograph presented in Figure 3(d) suggests that the long thin filopodia (<200 nm) are also entering the pore channels to enhance cell attachment, as indicated by the red arrows. Another feature seen in Figure 3(d) is the presence of numerous microvilli, which extended from the cell surface. Since the microvilli are actively involved in adsorption, cellular adhesion, and secretion [35], their presence indicates that the cells are interacting with both the ECM and the respective substrates.

**Figure 4:**
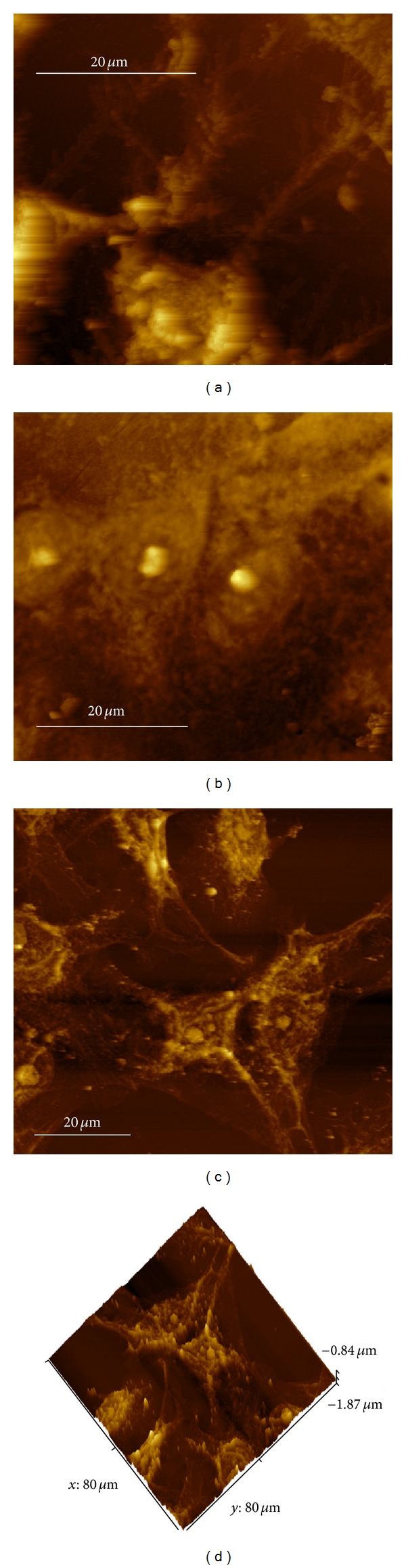
AFM images of cells and filopodia (a) glass substrate, (b) Whatman anodisc membrane, (c) in-house AAO membrane, and (d) angled view of in-house AAO membrane.

Despite not having the nanometre scale topographical features of the two nanoporous membranes, the glass substrate still promoted cell attachment over the entire surface. Examination of the optical microscopy images presented in Figures 2(a), 2(b), and 2(c) for 24 hours of cultivation confirmed surface coverage for all three substrates. In addition to the FESEM examination, an AFM study was carried out to confirm cell attachment.  Figure 4 presents the results of the AFM analysis of all three substrates. The cell images of all three substrates clearly show that the cells are firmly attached to the substrate and ECM. The AFM images reveal not only the cells but also the numerous filopodia extending from the cell. Of particular interest is the image presented in Figure 4(c), which clearly shows a number of filopodia extending over the surface of the in-house synthesised AAO membrane. An angled view of the same membrane is presented in Figure 4(d) and provides an enhanced view of the filopodia.

**Figure 5:**
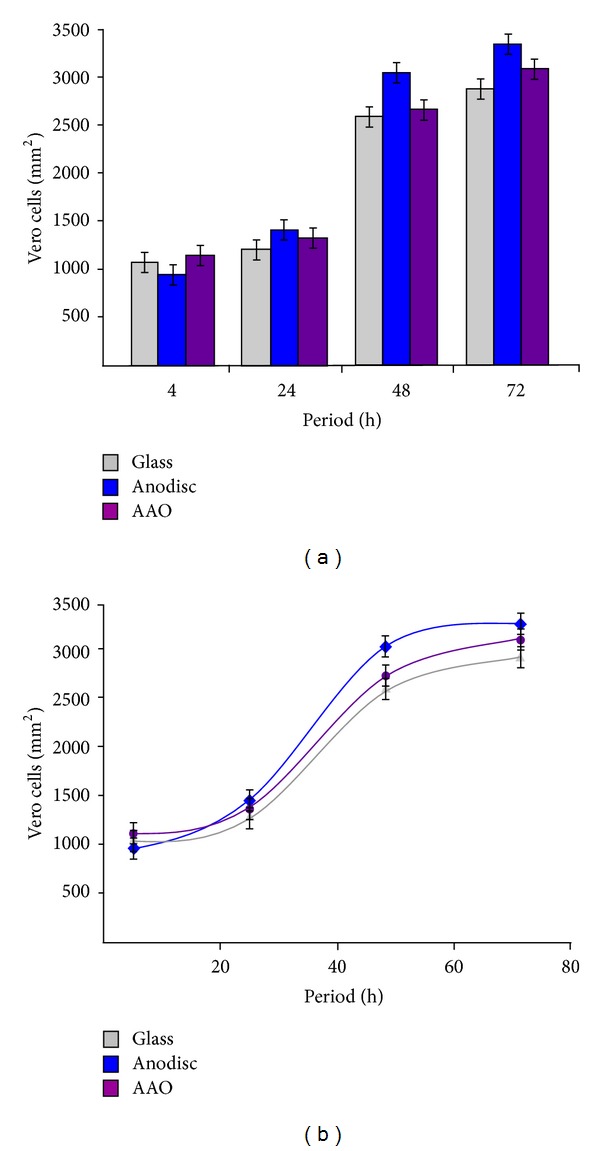
(a) Graphical presentation of the results of a 72 h cell proliferation study and (b) comparison between the in-house AAO membrane, Whatman anodisc membrane, and the glass control.

The present adhesion studies have confirmed that the Vero cells do indeed interact and attach to both nanoporous membranes and the glass control. However, the cell attachment study did not provide any biological information regarding the long-term survivability of the Vero cell line on the respective substrates. To address cell viability, a cell proliferation assay was undertaken since proliferation is an important factor in determining the long-term survival of cells on any tissue culture substrate. The results of the 72 h cell proliferation assay are presented in Figure 5. During the various assays there was no evidence of infection or toxicity effects occurring to any of the cells over the 72 h test period.

At the end of the first 4 h period, the number of cells adhering to the in-house AAO membrane was higher than both the glass control and the Whatman Anodisc membrane. The in-house AAO membrane recorded 11.6% more cells than the glass substrate, while the Anodisc membrane recorded 6.6% fewer cells than the glass substrate. As the assay progressed over the following days, the number of viable cells increased on all substrates. At the 24 h period, the number of viable cells on the Whatman Anodisc membrane was 14.9% greater than the glass control, while the in-house AAO membrane was 6.3% greater than the glass control. From this point on, the number of viable cells on the Whatman Anodisc membrane was always significantly greater than either the in-house AAO membrane or the glass control. By the end of the 48 h period, the number of viable cells on the Whatman Anodisc membrane was 16.2% greater than the glass control, while the in-house AAO membrane was only 4.1% greater than the glass control. And by the 72 h time period, the in-house AAO membrane was 8.8% greater than the glass control, while the Whatman Anodisc membrane recorded cells numbers 16.5% greater than the glass control. However, by the 72 h period, cells numbers on the Whatman Anodisc membrane had levelled out indicating that a confluent layer of cells had been achieved on the substrate. Also at this point a levelling trend in both the in-house AAO membrane and glass control could also be seen. At the end of the 72 h proliferation assay the highest number of viable cells was found on the Whatman Anodisc membrane, while the lowest number of viable cells was found on the glass substrate as shown in Figure 5(b).

A direct comparison between the in-house AAO membrane, the Whatman Anodisc membrane, and the glass control is presented in Figure 5(b). Inspection of Figure 5(a) reveals that, initially, the in-house AAO membrane had the largest number of viable cells attached to its surface. This was followed by the glass control and finally the Whatman Anodisc membrane. However, from the 20 h period onwards, the number of viable cells on the in-house AAO membrane is on average 6.4% greater than the glass control. While the number of viable cells on the Whatman Anodisc membrane is on average 15.8% greater than the glass control. The higher proliferation rate on the Anodisc membrane also results in the early formation of a confluent layer over the substrate surface. During the 72 h period, the overall proliferation rate was found to be slower for cells cultured on the glass control.

One factor that seems to be influencing cell behaviour and proliferation is the surface roughness of each substrate. In the case of the Vero cell line, the number of viable cells on the smoother glass control is lower when compared to the rougher surfaces of the nanoporous membranes. In the extreme case, the Whatman Anodisc membranes have numerous rough edges protruding up from the surface along the pore walls as shown in Figures 1(d) and 1(f). The roughness caused by these pore wall protrusions significantly increases the nanometre scale texturing of the membrane surface. This surface feature is not found on the in-house AAO membranes or on the glass controls. And as a result, by the end of the 72 h cell proliferation assay the number of viable Vero cells on the Whatman Anodisc membranes was 3.33 × 10^3^ cells/mm^2^, while the in-house AAO membranes had 3.11 × 10^3^ cells/mm^2^ and the glass control had 2.85 × 10^3^ cells/mm^2^. In the case of the Vero cell line, the study indicates that increasing surface roughness or texturing promotes greater cell proliferation. This suggests that it may be possible to influence Vero cell behaviour and proliferation by nanotexturing the surface of a membrane in a similar manner to the nanometre scale surface topography to the Whatman Anodisc membranes. The study also revealed that both the Whatman Anodisc membrane and the in-house AAO membrane are both capable of being used successfully as a cell culture substrate for the Vero cell line.

## 4. Conclusion

The present preliminary study confirms that it is indeed possible to culture the Vero cell line on nanoporous membranes. All membranes were used without further surface modifications and promoted both cell attachment and cell proliferation. Cell attachment to the membranes was confirmed by the presence of thin filopodia being used to anchor the cells to the nanometre scale pore structures. The membranes with greater surface roughness tended to promote greater numbers of viable cells. At the end of the 72 h cell proliferation assay, the number of viable cells found on the two types of nanoporous membranes confirmed that they were both superior to the glass control. Further studies are needed to investigate and clarify the effects of surface texturing on cell attachment and proliferation, since these factors are critical in establishing AAO membranes as a viable tissue scaffold for potential tissue engineering applications in the future.
